# Investigation of Copper-Less Gas Electron Multiplier Detectors Responses to Soft X-rays

**DOI:** 10.3390/s20102784

**Published:** 2020-05-14

**Authors:** Bartosz Mindur, Tomasz Fiutowski, Stefan Koperny, Piotr Wiącek, Władysław Dąbrowski

**Affiliations:** Faculty of Physics and Applied Computer Science, AGH University of Science and Technology, al. Mickiewicza 30, 30-059 Kraków, Poland; tomasz.fiutowski@agh.edu.pl (T.F.); stefan.koperny@fis.agh.edu.pl (S.K.); wiacek@agh.edu.pl (P.W.); wladyslaw.dabrowski@fis.agh.edu.pl (W.D.)

**Keywords:** micropattern gaseous detectors, gaseous imaging and tracking sensors, X-ray sensors

## Abstract

In this paper, we report on the systematic study of different variants of X-ray detectors based on Gas Electron Multiplier (GEM) technology using modified GEM foils with greatly reduced amount of copper. The main goal of this study was understanding the performance of such detectors applied in X-Ray Fluorescence (XRF) elemental analysis. Reduction of the amount of copper in the detector structure is crucial for suppression of XRF background from copper, but one has to ensure that key detector parameters are not affected by such modification. The tested detector variants include detectors with different types of copper-less GEM foils, which have been manufactured starting from standard copper-clad foils and removing partially the copper layer in additional post-processing steps. The results are analyzed and discussed with a particular focus on the energy resolution, uniformity of gas gain and energy resolution across the detector area, and on the long-term stability of the gas gain. Long-term stability tests performed for selected detectors do not indicate for any accelerated aging of the copper-less detectors compared to standard detectors using copper-clad GEM foils. The presented results lead us to conclude that the copper-less GEM detectors are promising devices to suppress the XRF background.

## 1. Introduction

Energy dispersive X-ray analysis and imaging is a well-known technique used in the non-destructive elemental analysis in many physical and chemical applications. Recently, the technique has been applied in a variety of other fields of studies not directly connected with basic research (e.g., [1,2]). Furthermore, this method has also been successfully implemented for the in-depth investigation of cultural heritage objects [3,4,5,6,7,8]. The GEM detectors are the most popular and widely used Micro-Pattern Gaseous Detectors (MPGD) [9,10,11,12], which are suitable for X-ray spectral imaging, despite their limited energy resolution [13,14]. They are very robust and rather easy to assemble from prefabricated components, with a variety of configurable options, allowing the sensor parameters to be adjusted to the specific requirements [15,16,17,18,19,20,21,22,23,24,25]. A standard GEM detector is assembled of copper-rich components. The X-ray fluorescence of copper present in the detector structure irradiated with primary photons of sufficiently high energy, above its absorption edge, results in significant background, which limits the sensitivity of elemental analysis, in particular for elements with characteristic radiation energies close to the copper K${}_{\alpha }$-line and K${}_{\beta }$-line of 8.05 keV and 8.90 keV, respectively [26]. Therefore, we propose a post-processing step for standard Cu-clad GEM foils to remove the copper cladding from most of the foil area, retaining only the adhesive chromium layer. Our initial measurements performed with copper-less GEM detector confirmed clearly that using copper-less GEM foils helps to suppress significantly the copper-originated fluorescence background [20].

**Figure 1:**
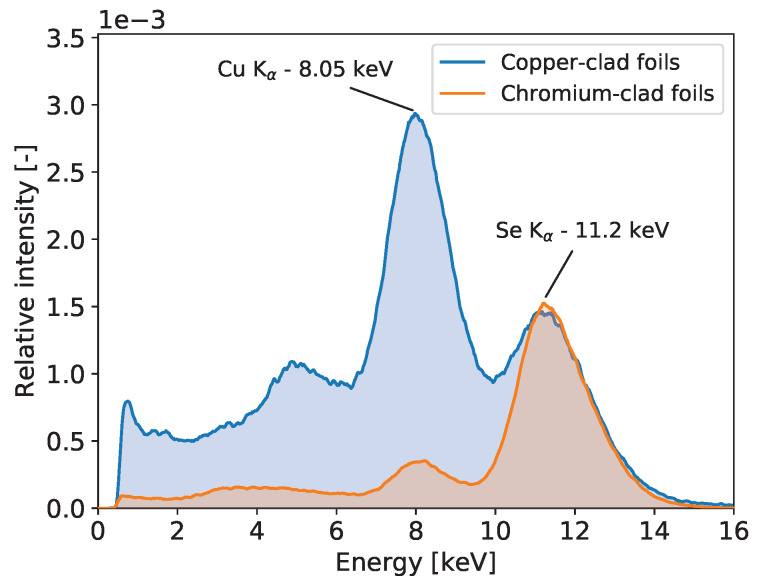
Spectra of selenium fluorescence obtained with the standard GEM detector with copper-clad foils and with the modified GEM detector using coper-less foils. One can observe large parasitic signal from excited copper-rich inner components of standard GEM detector in contrast to one with coper-less foils. The intensity is normalized to the area of the 11.2 keV peaks.

To illustrate the advantage of the detector with chromium-clad foils in XRF analysis, in comparison to the standard GEM detector, we measured the fluorescence radiation of selenium sample. The selenium K${}_{\alpha }$-line of 11.2 keV is close to the copper K${}_{\alpha }$-line and K${}_{\beta }$-line. Figure 1 shows the resulting spectra normalized to the area of the selenium peaks. In the case of coper-clad sensor, the parasitic copper fluorescence peak is much higher than the selenium one. Thus, for low amount of selenium to be detected, for example in an art object, the selenium signal may be lost completely in the copper background. The situation is much improved in the detector with Cr-clad foils where the copper peak and associated background are largely reduced.

In this paper, we report on systematic investigation of different variants of such copper-less detectors. The tested variants include detectors with different thicknesses of the chromium layer, detectors with different widths of the copper stripes, a detector with different pitches of the copper grid, and a detector with an aluminum drift electrode. Except the one with the aluminum drift electrode, in all other detectors, the chromium cladding and the copper grid on the drift electrode are the same as on the GEM foils for the given variant.

In [27], we reported on test results of a copper-less GEM detector using various gas mixtures. In the course of testing, we observed very significant degradation of the detector performance resulting in large variation of the gas gain across the detector, which occurred only after flushing the detector with a rather unconventional xenon and Trimethylamine (TMA) gas mixture. In this paper, we report on further systematic tests of GEM detectors using different types of copper-less GEM foils and different types of drift electrodes, but the same gas mixture Ar/CO${}_{2}$ 70/30. We focus on the energy resolution, uniformity of gas gain and energy resolution across the detector area, and on the long-term stability of the gas gain.

## 2. Materials and Methods

### 2.1. Copper-Less GEM Detectors

The detectors used for this investigation are based on the mechanical structure of the standard triple GEM detector with active area of 10 cm× 10 cm, but using various combinations of modified drift electrodes and GEM foils. The detector assembled in a gas-tight chassis, comprises low-mass kapton window, drift electrode, three GEM foils, and readout structure with two sets of orthogonal strips with a pitch of 400 µm. The GEM foils are patterned with holes arranged on a hexagonal grid with a pitch of 140 µm. The holes have double-conical cross-section with the inner diameter of 50 µm and the outer diameter of 70 µm. The drift region (volume between a drift foil and first GEM foil) has a thickness of 3 mm, while all other regions (transfer and induction) have a thickness of 2 mm. Such a construction of the GEM detector is very similar to the one developed for the COMPASS tracking detector [15].

The copper-less foils were manufactured starting from the standard foils with Cu cladding. The structure of such a standard foil includes a thin chromium adhesive layer underneath 5 µm copper layer. There are two types of such foils available on the market with different thickness of the chromium adhesive layer, either 10 or 100 nm. In the post-processing steps, the copper layers were removed from most of the area of the GEM and drift foils, leaving only the chromium layers and a grid of copper stripes to avoid electrical breaks in the case of development of cracks in the fragile chromium layer. A typical pitch of the copper grid is 1 cm. To investigate different grid options, special foils have been manufactured comprising four sections with different grid pitches. All the foils were produced by Technology Transfer Agency Techtra [28] and tested according to their standard procedures.

A complete list of the GEM detectors, which have been assembled and thoroughly tested is shown in Table 1. Detector numbering starts from $A_{2}$, as the first detector of this type ($A_{1}$) tested previously was significantly degraded after a test with a Xe/TME gas mixture [27].

Detector ($A_{2}$) is assembled of foils with 10 nm thin chromium layer and the copper grid with a pitch of 1 cm. Another variant of this detector type was equipped with a drift electrode with continuous aluminum layer instead of chromium one. This detector is listed as B in Table 1. Such an assembly is a first step towards a fully aluminum detector, i.e., equipped also with GEM foils with aluminum cladding. However, it is widely known that kapton foils with aluminum cladding are difficult to manufacture and handle due to fragility of the aluminum layer.

To verify that the 10 nm thin chromium layer provides sufficient surface conductivity, two other detectors assembled of foils with 100 nm thick chromium layers were tested. These are detectors $C_{1}$ and $C_{2}$ in Table 1.

**Figure 2:**
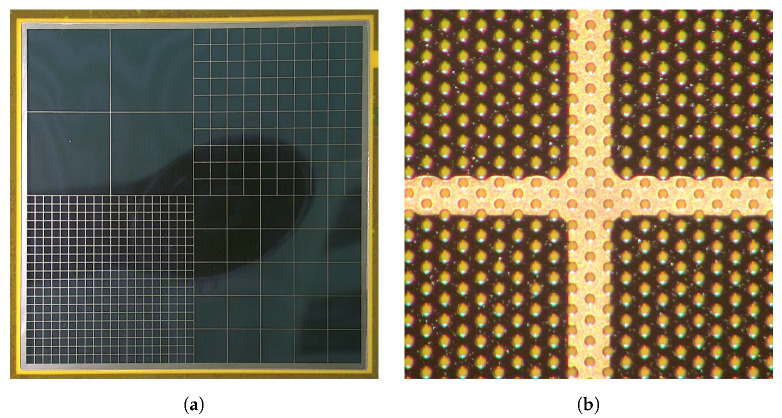
Pictures of the copper-less foils. (**a**) A photograph of copper-less GEM foil with different copper grid layouts; (**b**) A micro-photograph of the copper grid stripe (250 μm wide).

It is worth noting that the pitch of the copper grid of 1 cm was decided rather arbitrarily at the beginning of this development program and it appeared to work well. We have not experienced any problems with electrical continuity even for the foils with the thin chromium layer of 10 nm. To understand whether uniformity of the gas gain and energy resolution across the detector may depend systematically on the grid pitch, a set of foils comprising four quadrants with different grid pitch has been manufactured and a detector (D in Table 1) has been assembled from these foils and tested. Figure 2a shows a picture of the foil with different grids in four quadrants and Figure 2b shows the details of the grid structure. Finally, for comparison, we also include the test results for a detector using standard GEM and drift foils with copper cladding, which is the most commonly used structure (detector E in Table 1).

To decouple the effects associated with gas mixtures from the effects associated with the detector structure, all tests reported in this paper were performed using a standard mixture of Ar/CO${}_{2}$ (70/30) premixed and supplied by Air Liquide.

### 2.2. Test Set-Up and Test Procedures

A custom-designed test set-up capable of performing spectroscopic and imaging measurements was used to measure systematically performance of the detectors under evaluation. All measurements were done using the same front-end electronics and Data Acquisition (DAQ) system, gas supply system, detector high voltage bias supply, and voltage divider. The data taken for all tested detectors were analyzed using the same procedures. The detectors were mounted and tested in the set-up sequentially.

All tests were performed in a laboratory with temperature controlled by an air conditioning system, which maintained the temperature within a range of 3 °C. The gas pressure followed the atmospheric pressure with a slight overpressure. The gas flow rate was kept at a level of at least two detector volume exchanges per hour, which ensured that impurities out-gassing from detector inner components or gas supply system did not affect the tests.

Parameters and characteristics of the tested detectors were evaluated based on the measured spectra of ${}^{55}$Fe radioactive source, which allowed us to monitor the spatial distribution of the gas gain and of the energy resolution across the detector area. The source was placed above the detector plane at a distance of ∼20 cm to keep non-uniformity of the photon intensity across the detector area below a factor of two. The average photon count rate during the measurements was equal to 44 cps/mm2 over the whole sensor active area. The nominal activity of the ${}^{55}$Fe source was 7.4 GBq as at the beginning of 2019.

#### 2.2.1. Detector Readout System

The employed readout system is based on custom made Application Specific Integrated Circuits (ASICs) connected to a Field-Programmable Gate Array (FPGA) DAQ system. The ASIC, called GEMROC, was design using a 0.35 µm CMOS process [29]. It comprises 32 independent channels, each delivering the amplitude and time information for every signal with amplitude above a given discrimination threshold for the self-triggered operation mode. The timing channel is equipped with a discriminator, which delivers the signal to latch a 8 ns resolution time stamp and to enable the peak detect and hold circuit in the energy channel. The signal amplitude and the time stamp are stored in an analog and a digital de-randomizing buffers, respectively. The GEMROCs together with dedicated DAQ system offer a unique capability of simultaneous 2D position sensitive and spectroscopic measurements [30,31]. Thus, the system allows measuring the gas gain and energy resolution pixel by pixel. As a result, we obtain full 2D maps of the gas gain and energy resolution across the detector area. The maps of gas gain, energy resolution, and cumulative counts of events were reconstructed using a dedicated software suit [32] with a spatial resolution corresponding to pixel size of 800 µm × 800 µm.

#### 2.2.2. Measurement Procedure

**Figure 3:**
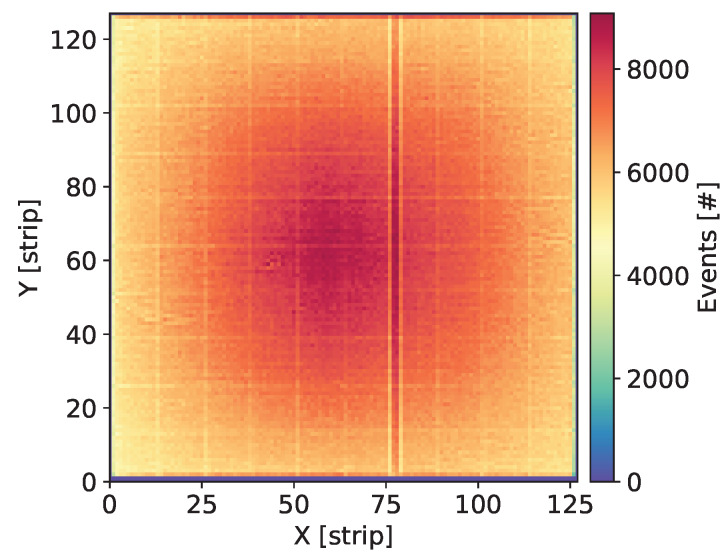
GEM detector $A_{2}$. Map of cumulative counts of reconstructed events for detector bias voltage of 3700 V.

Each newly assembled GEM detector was kept biased and flushed with the gas mixture for at least a few days before starting measurements. Systematic measurements were taken over a large range of the detector bias voltage in order to explore a wide range of the gas gain. Initial check of each detector was done by uniform irradiation of the whole detector area with X-rays from ${}^{55}$Fe source and visual inspection of the maps of cumulative counts, as shown in Figure 3, Figures 7, 11, 15 and 19. Such maps provide qualitative pictures of detector operation, possible faulty readout channels, or local defects in the detector itself.

#### 2.2.3. Gas Gain and Energy Resolution Maps Across Detector Area

For each detector bias voltage, the maps of the gas gain variation and energy resolution across the detector area are created. The charge generated in the avalanche is spread across a few readout strips of each coordinate resulting in clusters of signals is recorded in a group of strips. Every detected photon is assigned to a “pixel” defined by crossing of two orthogonal readout strips which have collected the largest signals. Following this step, the total cluster signal is calculated. To create full maps of the gas gain and energy resolution after event reconstruction, individual energy spectra are created for all detector “pixels”.

**Figure 4:**
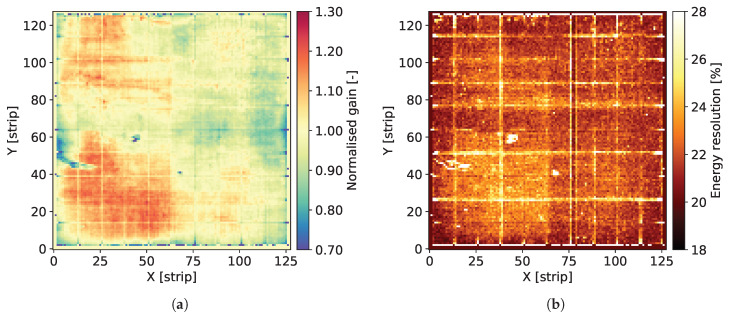
GEM detector $A_{2}$. 2D maps of: (**a**) normalized gas gain; and (**b**) energy resolution for detector bias voltage of 3700 V.

The individual spectra contain two well distinguished peaks corresponding to Mn K${}_{\alpha }$-line of 5.90 keV and the escape peak corresponding to energy of 2.94 keV. The signal amplitude corresponding to the 5.90 keV peak is used for estimation of the gas gain. The data corresponding to the peak area are fitted to a single Gaussian distribution providing the peak position and the standard deviation. For the peak positions, the average value for the whole detector is calculated and then the map of gas gain is normalized to this average level. As a result, we obtain maps of relative variation of the gas gain, such as the ones shown in Figure 4a, Figures 8a, 12a, 16a and 20a.

The Full Width at Half Maximum (FWHM) values of the 5.90 keV peaks are used as a measure of local energy resolution across the detector. It should be noted, however, that the energy resolution obtained by fitting the data to a single Gaussian peak is affected by the presence of the Mn K${}_{\beta }$-line of 6.49 keV. Since the statistics of recorded events for each individual pixel is relatively low, fitting these data to a distribution comprising two peaks is not reliable. Thus, maps such as the ones shown in Figure 4b, Figures 8b, 12b, 16b and 20b should be considered as illustration of the variation of the energy resolution across the detector area and not as a measure of the ultimate energy resolution of the detector.

#### 2.2.4. Detailed Analysis of Energy Spectra and Energy Resolution

Detailed analysis of the recorded spectra and extraction of the ultimate energy resolution is performed for the summed spectra across all pixels, which include much higher statistics allowing for more detailed analysis. The summed spectra are built after normalizing the gains of all pixels to the mean value. An example of such a spectrum for the entire detector after correction of the gas gain across the detector is shown in Figure 6. In the spectrum of the ${}^{55}$Fe radioactive source, we expect at least three peaks of energies 5.9, 6.49, and 2.94 keV corresponding to Mn K${}_{\alpha }$-line, Mn K${}_{\beta }$-line, and Ar escape peak, respectively. The dominant peak of 5.90 keV and the escape peak are clearly distinguishable in the spectra built with high statistics. However, one can notice that both peaks show visible distortion from perfect Gaussian distributions on the higher energy sides. In case of the 5.90 keV peak, this is due to presence of the Mn K${}_{\beta }$-line of 6.49 keV. In principle, one could expect also an Ar escape peak of 3.53 keV corresponding the 6.49 keV photons, but relative intensity of this second escape peak is too low to be distinguishable with a gaseous detector.

To extract the energy resolution for the 5.90 keV line, which is used as a common reference for comparison of various low energy X-ray spectrometry systems, the spectra such as the ones shown in Figures 6, 10, 14, 18 and 22 are fitted to a distribution comprising three Gaussian peaks corresponding to expected energies of 2.94 keV, 5.90 keV, 6.49 keV, and a continuous background, which is always present in GEM detectors due to scattering of photons in the detector material. The peaks drawn according to the fit parameters obtained for the 5.9 and 6.49 keV energy lines and the corresponding energy resolutions are shown in the plots.

#### 2.2.5. Gas Gain and Energy Resolution vs. Detector Bias Voltage

**Figure 5:**
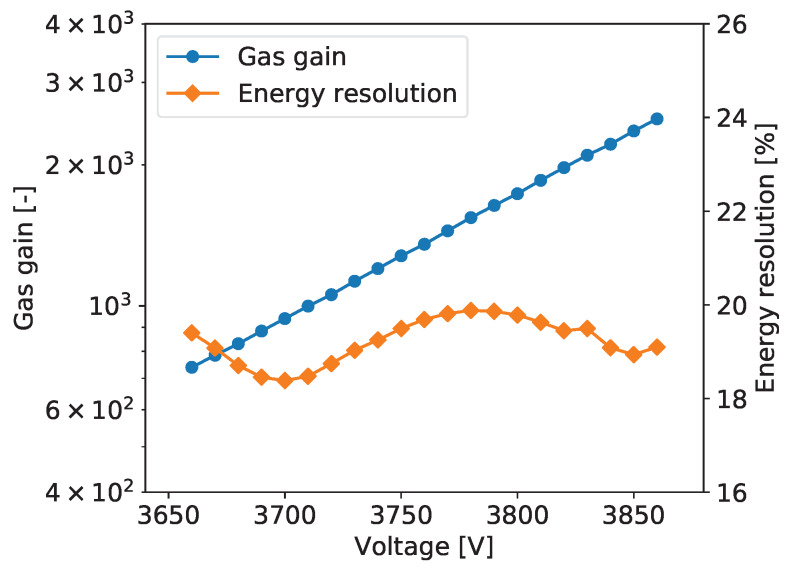
GEM detector $A_{2}$. Gas gain and energy resolution vs. detector bias voltage.

**Figure 6:**
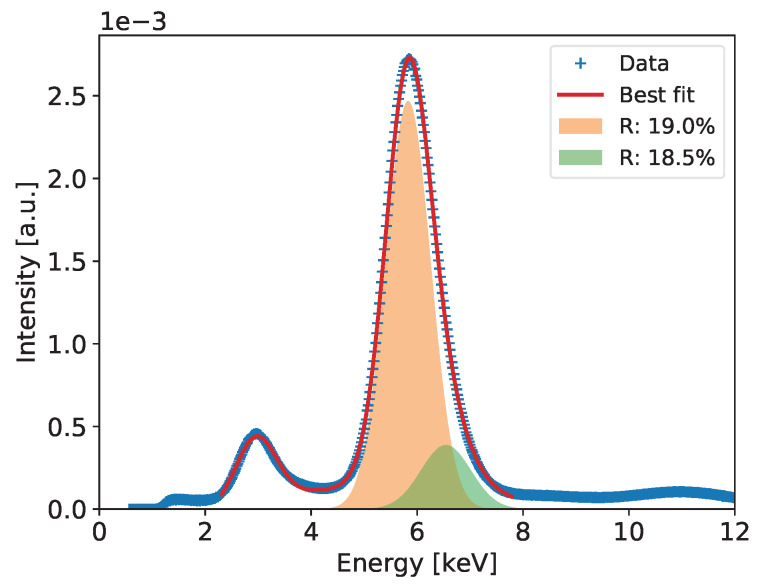
GEM detector $A_{2}$. ${}^{55}$Fe spectrum for detector bias voltage of 3700 V.

To explore performance of the tested detector as a function of the gas gain, measurements as described above were repeated for different detector bias voltages yielding the gas gain variation from about 600 to 3000. Figure 5 shows typical plots of the gas gain and energy resolution vs. detector bias voltage. The gas gain increases exponentially with increasing detector bias voltage, as expected. The plotted energy resolution values are the values for the 5.90 keV line extracted from the plots such as the one shown in Figure 6.

For all tested detectors, the energy resolution vs. detector bias voltage follows a characteristic tilde-like shape with a minimum and a maximum. A qualitative explanation of this dependence is as follows. For very low gas gain values the resolution is limited not only by fluctuations of the generated charge but also by the noise of the front-end electronics. Thus, with increasing gas gain, the signals increases and relative contribution of the electronic noise is reduced. As a result, the energy resolution reaches a minimum for a gas gain of about 1000. For higher gas gain values, a competitive effect becomes dominant, namely with increasing gas gain the fluctuations of the charge generated in avalanche multiplication processes increase resulting in worsening of the energy resolution. To explain further improvement of the energy resolution in the range of high gas gain, one should note that the measured spectra are built by summing the signals from a few strips within given cluster. Since the readout electronics is self-triggered using internal discriminators, some small signals on the cluster tails fall below the discrimination threshold resulting in additional fluctuations of the summed cluster signal. For higher signals generated in the detector the fractions of electronic signals missed in the clustering procedure become smaller and we observe improvement of the energy resolution. However, exploration of higher gas gain region is limited by the dynamic range of the readout electronics, which is based on multichannel integrated circuits, in which the electronic gain is tunable only within a limited range.

#### 2.2.6. Long Term Stability of the Gas Gain

Three selected GEM detectors ($A_{2}$, $C_{1}$, and E in Table 1) were used for long-term stability measurements. The test procedures in all cases were very similar, i.e., for a given bias voltage, for which the detector response was already known, we recorded continuously the signals generated by photons from the ${}^{55}$Fe source. The measurements were continued for at least one month period without any interruption, for each detector. Within this period, most of the operation conditions were kept constant. The only changes were due to the atmospheric pressure fluctuations and ambient temperature variations, which were not compensated by the air conditioning system in the lab. The results are shown in Figure 23a–c.

## 3. Results and Discussion

### 3.1. GEM Detector with 10 nm Chromium-Clad Foils

In this section, we present the results for the GEM detector $A_{2}$. It is equipped with similar GEM foils as the one described in [27], i.e., the foils have thin 10 nm chromium layers. Figure 3 shows the map of cumulative counts of reconstructed events. One can notice grid lines with lower intensity corresponding to the grid of copper stripes reinforcing the drift electrode and the GEM foils. As demonstrated below by comparison with a detector with Al-clad drift electrode, loss of intensity is primarily caused by absorption in the copper grid on the drift electrode. The single vertical line with higher intensity is caused by a defective strip in readout structure and can be ignored in the discussion of global parameters of the tested detectors.

Figure 4a shows the map of normalized gas gain and Figure 4b shows the map of energy resolution obtained from course fitting of the 5.90 keV peaks to a single Gaussian distribution. The map shown in Figure 4a is typical for triple GEM detectors. Distribution of the gas gain values has a rather random pattern with smooth transitions between regions of different gas gain. The range of gas gain spread is similar to what we observed in previously tested detectors. One can notice the traces of lower gas gain corresponding to the copper grid, which indicate that the copper layer affects the electric field profiles inside the holes of the GEM foils. However, the range of these local variation of the gas gain correlated with the presence of copper is within the range of global variation of the gas gain across the entire detector, which very likely is due to non-uniformity of hole parameters across the GEM foils. Thus, aiming at applications requiring a high performance detector, pixel by pixel off-line correction of the gas gain will be mandatory.

Figure 4b presents the map of energy resolution. Here, the traces of the copper grid are more distinctive. The energy resolution is worse in the areas covered by the copper stripes. Visual comparison of the maps of gas gain and energy resolution leads to an observation that the two parameters are correlated, in particular along the copper stripes. However, the relative variation of the energy resolution (±22%) is smaller than the relative gain variation (±30%).

The plots of gas gain and energy as a function of the detector bias are shown in Figure 5. The gas gain increases exponentially with the detector bias, as expected, while the energy resolution vs. detector bias voltage characteristic has the typical tilde-like shape, as discussed in Section 2.2.5. The energy resolution varies in the range from 18% to 20%. The measurements were taken at an average counting rate of 54 cps/mm^2^.

Figure 6 shows the measured spectrum for the ${}^{55}$Fe radioactive source with a Gaussian two-peak fit to the regions of the 5.9 and 6.49 keV peaks. The red curve shows the global fit, while with different color fills each peak is separately visualized to show details of the fitting results. In the figure, the values of energy resolution for the two peaks are also shown.

### 3.2. Detector with 10 nm Chromium-Clad GEM Foils and 5 µm Aluminum-Clad Drift Electrode

In this section, we present the results for the GEM detector B. It is equipped with similar GEM foils like the ones described in [27] but with a different drift electrode. The drift electrode is made of a kapton foil one side covered with 5 µm aluminum layer. Using an aluminum drift electrode is motivated by the same reason the copper-less electrode with copper grid, i.e., to decrease further amount of copper and consequently X-ray fluorescence from inner detector components. Unfortunately, at that stage of the development, aluminum-based GEM foils were not available, therefore only the aluminum drift electrode was used.

**Figure 7:**
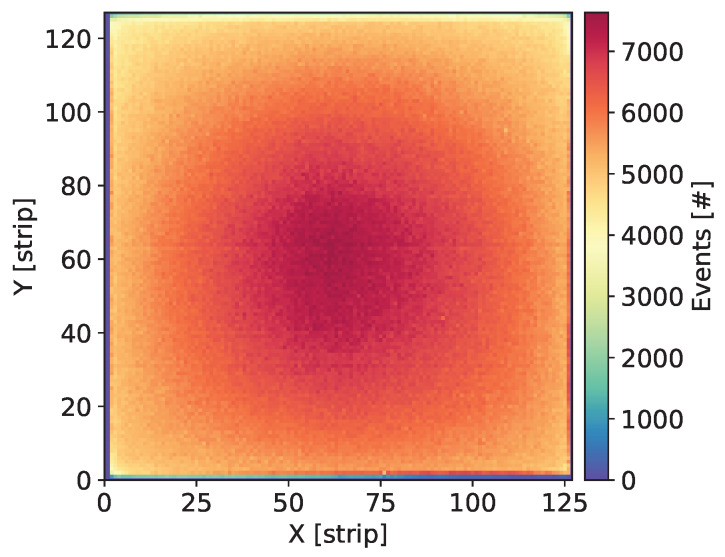
GEM detector B. Map of cumulative counts of reconstructed events for detector bias voltage of 3760 V.

Figure 7 shows the map of cumulative counts of reconstructed events. The measurements were taken at an average counting rate of 47.4 cps/mm^2^. One can notice a smooth distribution of intensity which is expected given that there is no grid structure in the aluminum drift electrode.

**Figure 8:**
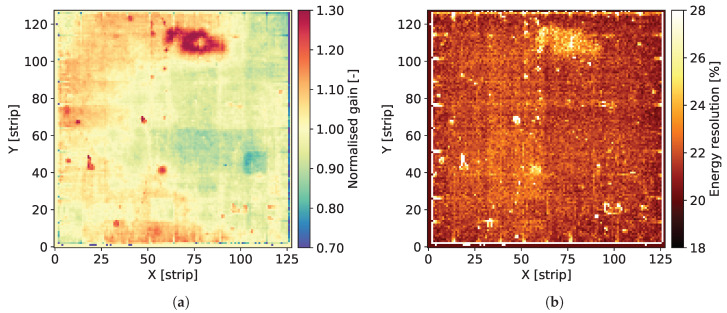
GEM detector B. 2D maps of: (**a**) normalized gas gain; and (**b**) energy resolution for detector bias voltage of 3760 V.

Figure 8a,b show the map of normalized gas gain and the map of energy resolution, respectively, obtained from course fitting of the 5.90 keV peak to a single Gaussian distribution. The map shown in Figure 8a is typical with a rather random pattern; however, a few “hot-spots” are clearly visible. Such high gain spots were observed only for this particular detector. They may be due local variation of processing conditions, such as etching, leading to local variation of parameters of holes in the GEM foils.

**Figure 9:**
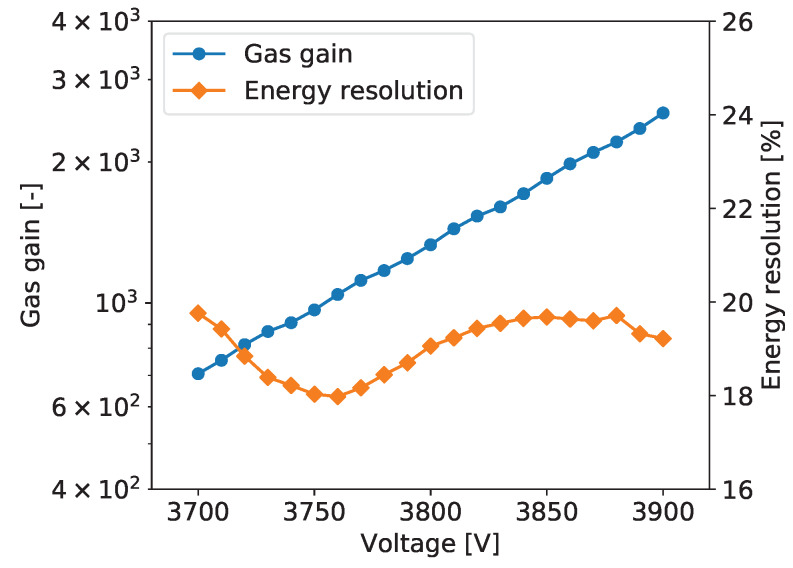
GEM detector B. Gas gain and energy resolution vs. detector bias voltage.

**Figure 10:**
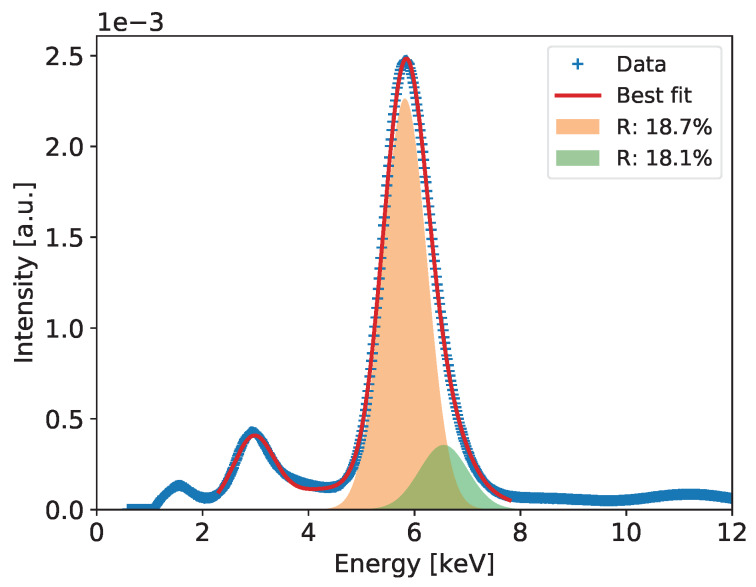
GEM detector B. ${}^{55}$Fe spectrum for detector bias voltage of 3760 V.

The plots of gas gain and energy resolution as a function of the detector bias are shown in Figure 9. The results are very similar to those described in Section 3.1. One can see a typical increase of the gas gain with increasing high voltage, while the energy resolution varies from 18% to 20%. Figure 10 shows the measured spectrum for the ${}^{55}$Fe radioactive source with a Gaussian two-peak fit to the regions of the 5.90 and 6.49 peaks. The energy resolution is slightly better than for detector $A_{2}$.

### 3.3. GEM Detector with 100 nm Chromium-Clad Foils

Two detectors, $C_{1}$ and $C_{2}$, using the GEM foils with 100 nm thick chromium cladding were assembled and tested. Foils with thick chromium cladding were used as we were afraid of possible problem with mechanical integrity of a thin chromium layer, which in the end appeared to be not a serious problem if the foils were handled carefully. The thickness of a chromium adhesive layer does not affect performance of a standard copper-clad GEM foils-based detector, in which the foil material is dominated by the topmost copper layer.

**Figure 11:**
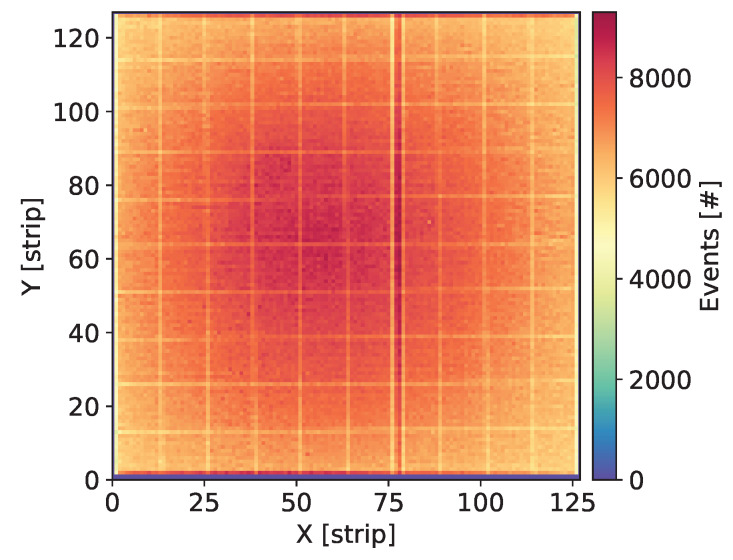
GEM detector $C_{2}$. Map of cumulative counts of reconstructed events for detector bias voltage of 3840 V.

The characteristics of these two detectors are very similar, thus we present only test results for detector $C_{2}$. A map of cumulative counts of reconstructed events is shown in Figure 11. It was obtained for a detector bias voltage of 3850 V and average rate of the photons of 35.2 cps/mm^2^. One can see clearly again the grid of lower intensity corresponding to the grid of copper stripes. The pattern is the same as for all other detectors using chromium-clad drift electrode. In addition, one can notice slightly higher intensity for one strip, which again can be ignored when considering the basic detector characteristics.

**Figure 12:**
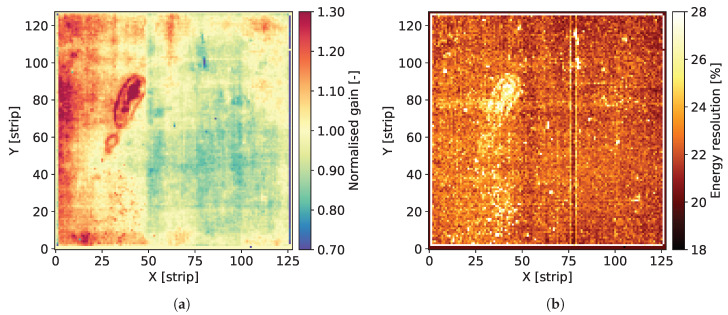
GEM detector $C_{2}$. 2D maps of: (**a**) normalized gas gain; and (**b**) energy resolution for detector bias voltage of 3840 V.

**Figure 13:**
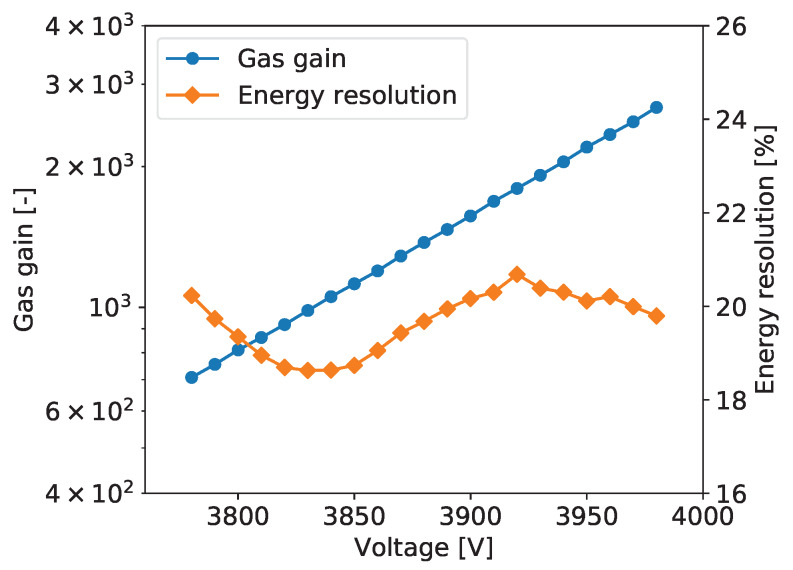
GEM detector $C_{2}$. Gas gain and energy resolution vs. detector bias voltage.

**Figure 14:**
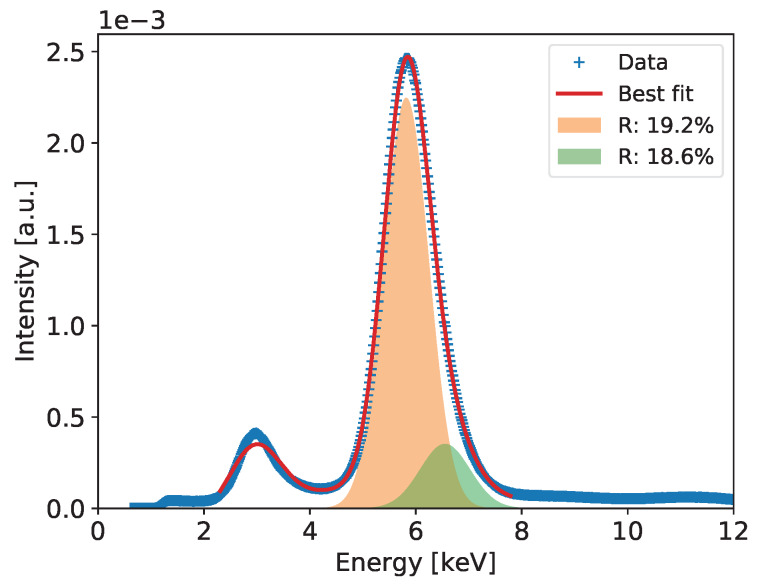
GEM detector $C_{2}$. ${}^{55}$Fe spectrum for detector bias voltage of 3840 V.

Figure 12a,b show 2D maps of normalized gas gain and energy resolution across the detector, respectively. The detector has typical gas gain fluctuations across its active area with exception of one spot (around coordinates [40, 85] in Figure 12a) with higher gain. This again indicates that the foil processing parameters are not perfectly uniform across the foil area. One can notice a weak correlation between the maps of gas gain and energy resolution in Figure 12b. The plots of gas gain and energy resolution as a function of the detector bias are shown in Figure 13. The results are very similar to those obtained for other detectors. Figure 14 shows the spectrum of the ${}^{55}$Fe radioactive source with a Gaussian two-peak fit to the regions of the 5.9 and 6.49 keV peaks. The energy resolution is slightly worse than for detector $A_{2}$ equipped with GEM foils with thin chromium cladding.

### 3.4. GEM Detector with 100 nm Chromium-Clad Foils with Various Copper Grid Layouts

As mentioned above, at the beginning of this program of systematic studies, arbitrarily selected pitch of the copper grid of 1 cm was used. Given that the primary goal of this exercise was to reduce the amount of copper in the detector structure, an obvious question was whether detector performance might be affected by the pitch of the copper stripes. Thus, we designed and manufactured copper-less GEM foils and drift electrode with 100 nm chromium cladding divided into four different sections with different pitches of the copper grid: 25, 10, 5, and 2.5 mm, as shown in Figure 2a. One can treat each quadrant of the detector as (almost) independent GEM detector of active area 50 mm × 50 mm.

**Figure 15:**
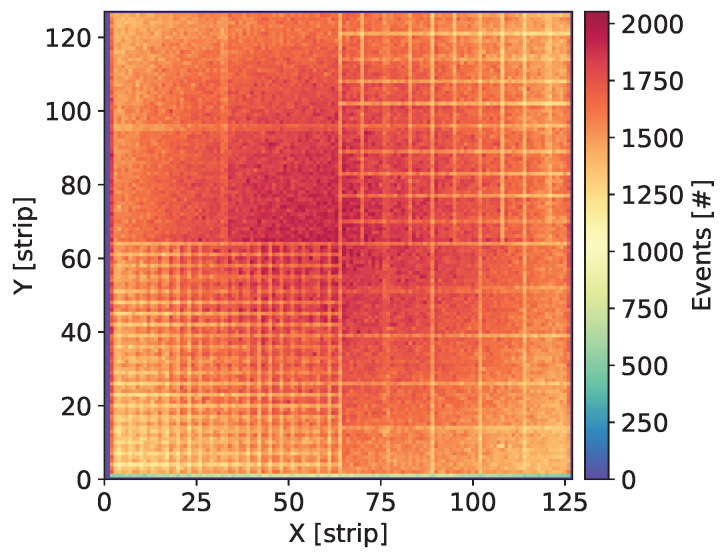
GEM detector D. Map of cumulative counts of reconstructed events for detector bias voltage of 3770 V.

The detector was tested following the same test procedure as applied for all other detectors described in this paper. An average global counting rate during the measurement was equal to 38.6 cps/mm^2^. The map of cumulative counts of reconstructed events is shown in Figure 15. The intensity pattern reveals clearly the patterns of the copper grid in the four sections of the detector, which is an obvious result.

**Figure 16:**
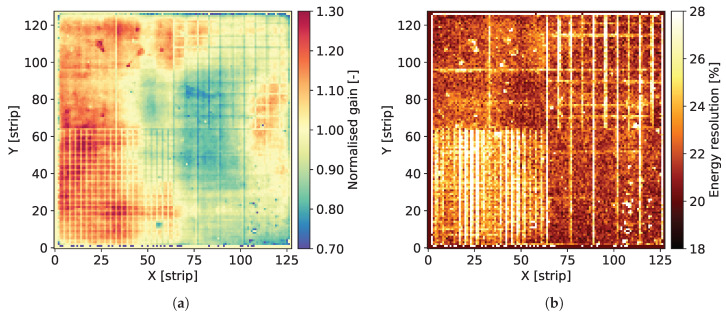
GEM detector D. 2D maps of: (**a**) normalized gas gain; and (**b**) energy resolution for detector bias voltage of 3770 V.

The grid pattern is also visible on the maps of gas gain and energy resolutions shown in Figure 16a,b, respectively. The local variations of gas gain along the grid lines are, however, smaller than the global variation across the entire detector surface. On the other hand, the density of the copper stripes affects the average energy resolution for the given section.

**Figure 17:**
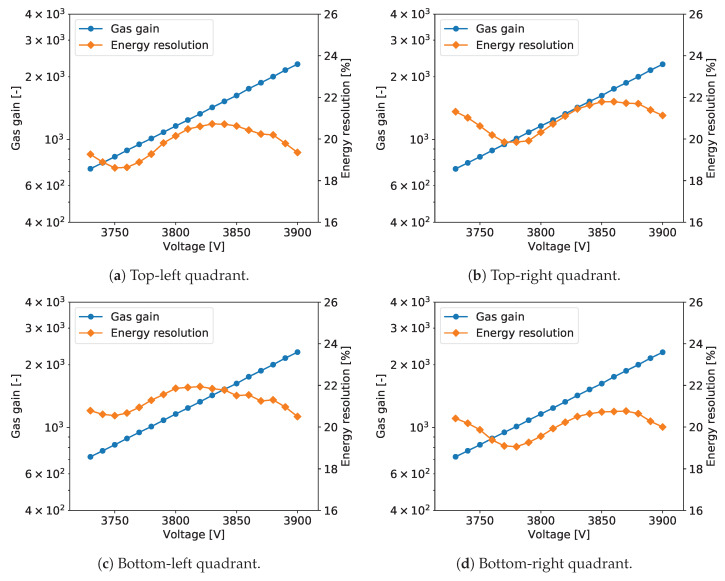
GEM detector D. Gas gain and energy resolution vs. detector bias voltage for each quadrant of the detector.

**Figure 18:**
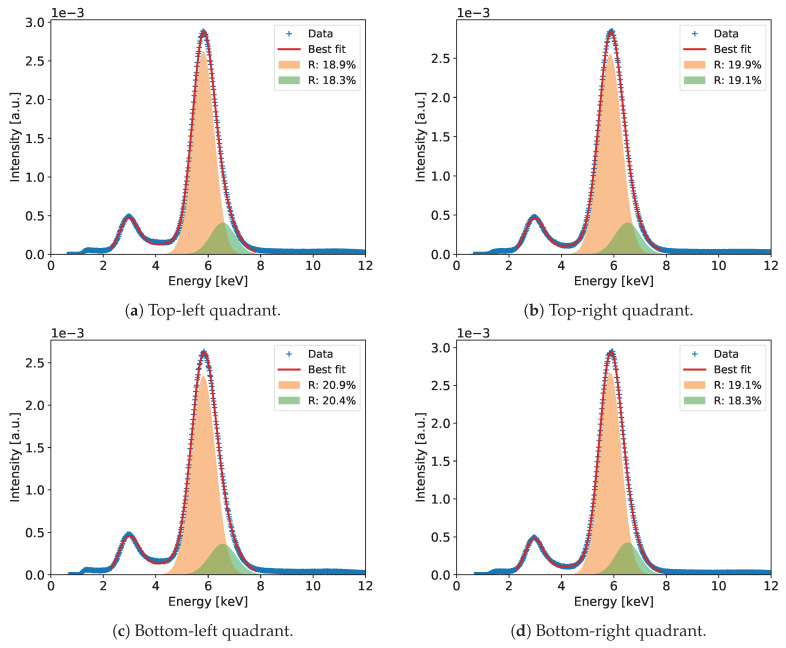
GEM detector D. ${}^{55}$Fe spectrum for detector bias voltage of 3770 V for each quadrant of the detector.

The plots of gas gain and energy resolution as a function of the detector bias voltage for the four quadrants of detector D are shown in Figure 17. The differences in the gas gain between the four sectors are small and almost not noticeable in log scale. However, one can notice differences in the energy resolutions, although the profiles of energy resolution vs. bias voltage are similar for all detector sections. The corresponding ${}^{55}$Fe spectra with extracted energy resolutions for 5.90 keV are shown in Figure 18. The best energy resolution of 18.9% for 5.90 keV is obtained for the section with only two crossing copper stripes, while the section with the highest density of copper strips shows the worst energy resolution of 20.9% for 5.90 keV.

### 3.5. GEM Detector with Standard Copper-Clad Foils

**Figure 19:**
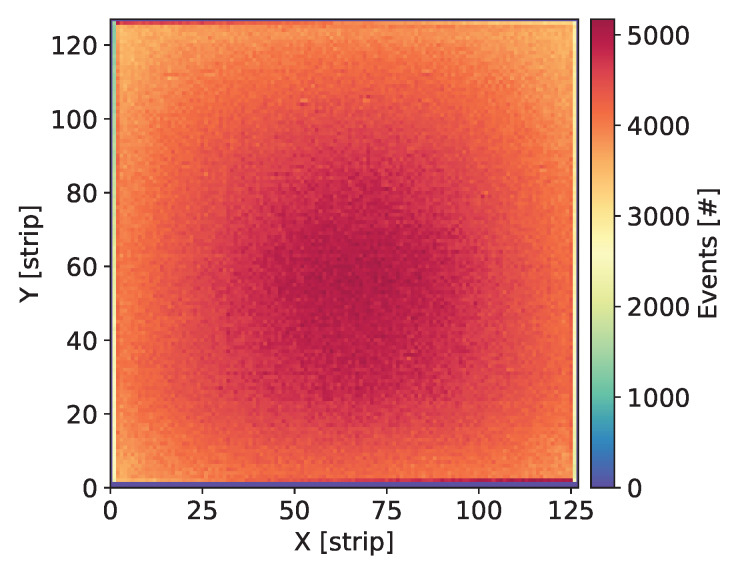
Standard GEM detector E with copper foils. Map of cumulative counts of reconstructed events for detector bias voltage of 3810 V.

To verify that the observed characteristics of various copper-less detectors are not affected by the overall system performance including the gas supply system, detector bias and voltage divider circuit, readout electronics, and data analysis procedure, a standard GEM detector using copper-clad foils was assembled and tested. A map of cumulative counts of reconstructed events for detector E for average count rate of 34.7 cps/mm^2^ is shown in Figure 19.

**Figure 20:**
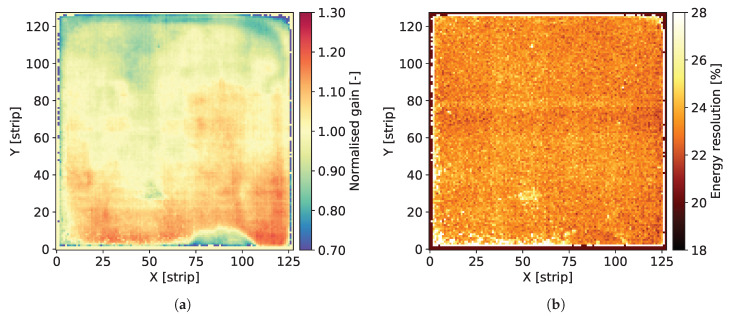
Standard GEM detector E with copper foils. 2D maps of: (**a**) normalized gas gain; and (**b**) energy resolution for detector bias voltage of 3810 V.

Figure 20a,b show 2D maps of gas gain and energy resolution across the detector, respectively. The range of variation of the gas gain is similar to that observed for the copper-less detectors; however, the map is rather smooth without significant local variations, compared to the maps obtained for copper-less detectors. This observation may indicate that the non-uniformity of the gas gain distribution in copper-less detectors are introduced in the additional processing step of etching the copper layer.

**Figure 21:**
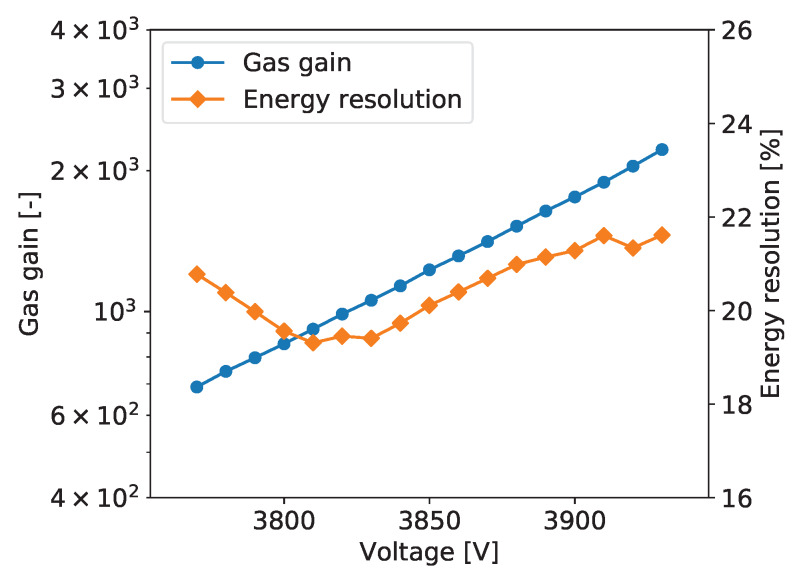
Standard GEM detector with copper foils E. Gas gain and energy resolution vs. detector bias voltage.

**Figure 22:**
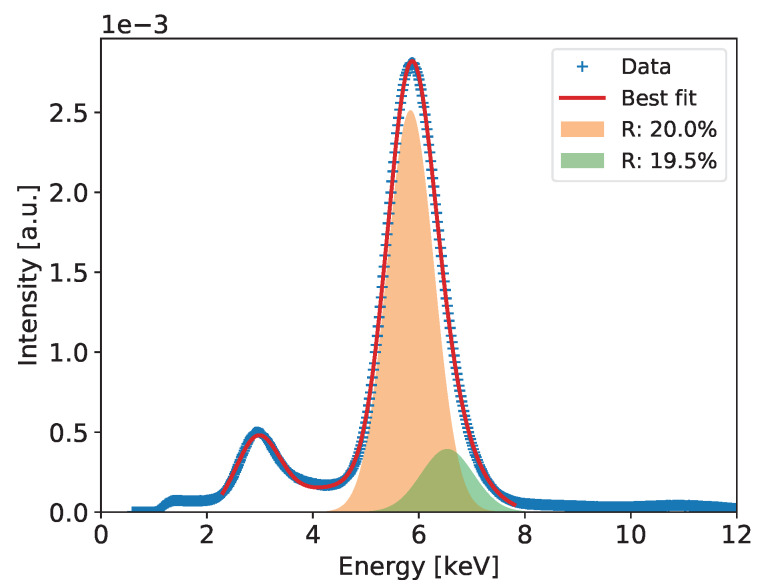
Standard GEM detector E with copper foils. ${}^{55}$Fe spectrum for detector bias voltage of 3810 V.

The plots of gas gain and energy resolution as a function of the detector bias are shown in Figure 21. The profiles are similar to those obtained for other detectors, although the energy resolution for this detector is slightly worse compared to the copper-less detectors. The spectrum of ${}^{55}$Fe radioactive source shown in Figure 22 confirms that the average energy resolution for the optimal bias conditions is also worse compared to the copper less detector, despite quite uniform distribution of the energy resolution across detector area (cf. Figure 20b).

### 3.6. Long-Term Studies of Selected Detectors

In our first report on copper-less GEM detector performance [27], we addressed the issue of significant gas gain degradation in time and development of spectacular mosaic patterns in 2D maps of gas gain. Then, we concluded that the observed accelerated aging of the detector was most likely caused by the TMA admixture used at a certain step of testing. Thus, to verify that our conclusion was correct and that the copper-less detectors are not intrinsically sensitive to aging processes, in addition to the tests reported above, we performed long-term tests for two copper-less detectors, $A_{2}$ and $C_{1}$, and for standard detector E.

The detectors were measured one after the other in series using the same readout, DAQ, and gas supply systems. The only parameters that varied during the tests were ambient temperature, atmospheric pressure, and humidity (not monitored). Each test lasted at least a thousand hours (six weeks). An average photon rate during all these measurements was similar, at a level of 35–54cps/mm^2^.

**Figure 23:**
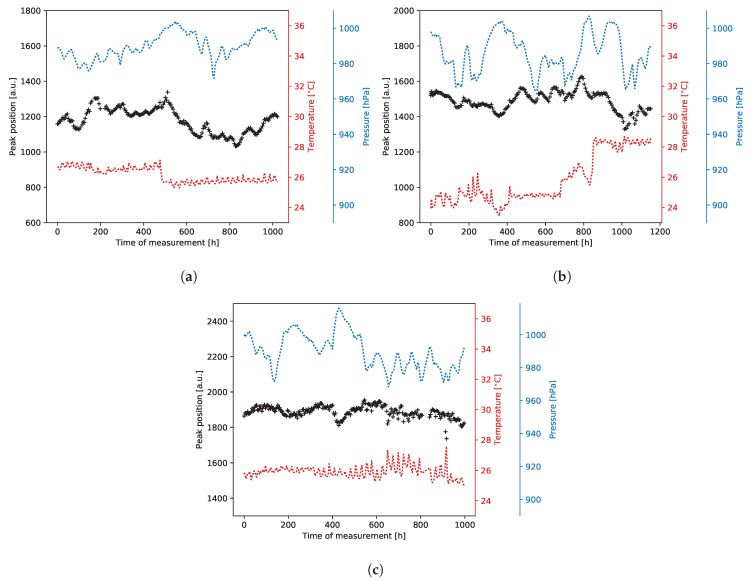
Long-term measurements results. (**a**) GEM detector with 10 nm chromium foils ($A_{2}$); (**b**) GEM detector with 100 nm chromium foils ($C_{1}$); (**c**) Standard GEM detector with copper foils (E).

The results of the studies are presented in Figure 23. Each plot (Figure 23a–c) shows the 5.90 keV peak position, detector temperature and atmospheric pressure as a function of time. At first glance, all three plots look very similar without indications of systematic decrease of the gas gain (represented by peak position) in time. These results confirm that the accelerated aging reported in [27] was not intrinsically associated with the structure of copper-less GEM foils.

## 4. Conclusions

Systematic studies of various types of copper-less detectors yield quite consistent results allowing us to conclude that characteristics of such detectors in terms of gas gain and energy resolution vs. detector bias voltage are not different from characteristics of standard detectors using copper-clad GEM foils. A copper grid on the foils results in additional spatial fluctuations of the gas gain and energy resolution; however, these fluctuations are smaller than the global variation of gas gain and energy resolution across the whole detector area. Since high performance characteristics of GEM detectors with energy resolution below 20 % for 5.90 keV can be achieved only by off-line correction of the gas gain on a pixel basis, these local variations of the gas gain are corrected in the same way as global variations of the gas gain. The energy resolutions obtained for the tested detectors are summarized in Table 2.

Comparison of characteristics of different sections of detector D suggest that one can consider using chromium-clad foil with greatly reduced reinforcing copper grid. It is worth noting that the detector section with only two crossing lines shows the best energy resolution.

The long-term tests confirmed stable operation over reasonably long periods of time, although the behavior of such copper-less detectors on the years time scale remains an open question. Similarly, aging of such detectors as a function of total accumulated charge needs to be tested; however, such tests can only be performed on much longer time scale than the test reported in this paper.

In summary, we conclude that the technology of copper-less GEMs foils is a promising one for manufacturing detectors for applications in which Cu fluorescence radiation background is a critical issue. Of course, more data are needed on reliability and production yield of such detectors based on higher statistics. In addition, safe procedures of handling copper-less GEMs foils need to be worked out.

## Figures and Tables

**Table 1:** List of assembled and tested detectors.

Detector Label	Drift Type	GEM Foil Type
$A_{2}$	Cr 10 nm	Cr 10 nm
B	Al 5 µm	Cr 10 nm
$C_{1}$	Cr 100 nm	Cr 100 nm
$C_{2}$	Cr 100 nm	Cr 100 nm
D	Cr 100 nm	Cr 100 nm
E	Cu 5 µm	Cu 5 µm

**Table 2:** Average energy resolution for selected detectors.

Detector	Energy Resolution for 5.90 keV [%]
$A_{2}$	19.0
B	18.7
$C_{2}$	19.2
D ${}_{\mathrm{BL}}$	20.9
D ${}_{\mathrm{TL}}$	18.9
D ${}_{\mathrm{BR}}$	19.1
D ${}_{\mathrm{TR}}$	19.9
E	20.0

## Abbreviations

The following abbreviations are used in this manuscript:

**ASIC**	Application Specific Integrated Circuit
**DAQ**	Data Acquisition
**FPGA**	Field-Programmable Gate Array
**FWHM**	Full Width at Half Maximum
**GEM**	Gas Electron Multiplier
**MPGD**	Micro-Pattern Gaseous Detectors
**TMA**	Trimethylamine
**XRF**	X-Ray Fluorescence
